# Lignocellulosic ethanol production by starch-base industrial yeast under PEG detoxification

**DOI:** 10.1038/srep20361

**Published:** 2016-02-03

**Authors:** Xiumei Liu, Wenjuan Xu, Liaoyuan Mao, Chao Zhang, Peifang Yan, Zhanwei Xu, Z. Conrad Zhang

**Affiliations:** 1 State Key Laboratory of Catalysis, Dalian National Laboratory for Clean Energy, Dalian Institute of Chemical Physics, Chinese Academy of Sciences, Dalian 116023, P. R. China

## Abstract

Cellulosic ethanol production from lignocellulosic biomass offers a sustainable solution for transition from fossil based fuels to renewable alternatives. However, a few long-standing technical challenges remain to be addressed in the development of an economically viable fermentation process from lignocellulose. Such challenges include the needs to improve yeast tolerance to toxic inhibitory compounds and to achieve high fermentation efficiency with minimum detoxification steps after a simple biomass pretreatment. Here we report an *in-situ* detoxification strategy by PEG exo-protection of an industrial dry yeast (starch-base). The exo-protected yeast cells displayed remarkably boosted vitality with high tolerance to toxic inhibitory compounds, and with largely improved ethanol productivity from crude hydrolysate derived from a pretreated lignocellulose. The PEG chemical exo-protection makes the industrial *S*. *cerevisiae* yeast directly applicable for the production of cellulosic ethanol with substantially improved productivity and yield, without of the need to use genetically modified microorganisms.

Cellulosic ethanol from lignocellulosic biomass through biological fermentation has been recognized as a sustainable transportation fuel due to the most abundant carbohydrate content of the broadly distributed non-food feedstock^1^,^2^,^3^. Very high gravity (VHG) fermentation, referring to the fermentation of high sugar concentrations, offers the advantages of improved overall ethanol productivity (producing ethanol in 10–15 vol%), reduced capital cost, and reduced energy input compared to processes at normal gravity^4^. This technology represents a major progress toward cost-competitive production of cellulosic ethanol. With lignocellulosic biomass as the feedstock, a pretreatment process is typically necessary to generate monomeric sugars from the polysaccharide components of the biomass for the subsequent fermentation process. However, typical pretreatment processes of lignocellulosic materials inevitably generate degradation compounds, e. g., acetic and formic acids, furfural, and 5-hydroxymethylfurfural (HMF) and phenolic compounds^5^,^6^,^7^. The residue of these compounds often exists in fermentation broth and functions as toxic inhibitors^8^,^9^,^10^. To achieve fermentable sugars in a high-concentration for VHG fermentation, the biomass loading ratio during pretreatment must be increased to a considerably high level, which typically results in high concentrations of inhibitors in the fermentation broth. These inhibitors often significantly reduce the rates of yeast metabolism and the final ethanol titers in the subsequent fermentation step^11^,^12^. The detrimental effect of the inhibitors remains one of the major barriers to the development of an economically viable process for cellulosic ethanol production^13^,^14^,^15^.

To overcome the issues related to the inhibitory compounds in the lignocellulosic hydrolysates, some techniques on detoxifying the hydrolysates by removing the toxic chemical residues have been reported, including physical (evaporation and membrane separation), chemical (over-liming, activated charcoal treatment, ion exchange, neutralization and organic solvent extraction), biological (treatment with laccase or peroxidase)^16^,^17^,^18^,^19^,^20^,^21^,^22^. However, these additional detoxification steps increase the overall costs due not only to the capital and chemical costs, but also to the loss of sugars^23^.

To reduce the cost associated with the detoxification steps, one potential solution is to develop fermentation microorganisms that are more tolerant to high concentrations of inhibitors in the lignocellulosic hydrolysates. The recent U.S. Department of Energy’s research roadmap^24^ highlighted a number of challenging targets, including increasing the tolerance of microorganisms to inhibitors present in hydrolysates. Strategies such as yeast adaptive evolution^25^,^26^, genetic engineering^27^, and evolution engineering^28^,^29^ have been used to develop more tolerant strains with improved fermentation capability for lignocellulosic hydrolysates without extra detoxification steps. Although attractive in this respect, the performance of laboratory strain is generally weak under the harsh conditions found in industrial fermentations. Moreover, the use of recombinant yeast strains increases production costs as well as biological risks^30^.

Our previous study^31^ showed that fully water-soluble polyethylene glycol (PEG) boosts the ethanol fermentation performance of industrial dry yeast (starch-base) cells in VHG media. The PEGs improved the vitality of the yeast cells under high glucose and ethanol concentrations in the absence of toxic compounds.

In this work, the addition of PEGs to a lignocellulosic hydrolysate fermentation broth was found to induce a highly favourable effect in vitalizing the yeast cells, resulting in substantially enhanced cell tolerance to toxic inhibitory compounds and largely improved ethanol productivity. To the best of our knowledge, the capability of PEGs to protect yeast cells from the toxicity of the inhibitors in lignocellulosic hydrolysates for ethanol production has not been reported. In terms of ethanol production from lignocellulosic feedstock, this finding leads a new strategy that offers three major potential economic benefits. First, it removes extra steps to detoxify the lignocellulosic hydrolysate by purifying the sugar solutions. Second, industrial dry yeast (starch-base) can now be used because the performance of the yeast cells exo-protected by PEGs becomes unabated in the presence of toxic inhibitors. Third, the fermentation process can now be applied for ethanol production from a lignocellulosic biomass simply after a low cost hydrolysis combined with steam explosion. A poplar sample is used in this work to demonstrate high ethanol productivity without additional detoxification steps following hydrolysis and steam explosion pretreatment.

## Results

### Toxicities of phenol, guaiacol, furfural, levulinic acid, HMF and vanillin on ethanol production

In order to assess the effect of the major types of toxic compounds present in the lignocellulosic hydrolysate on ethanol production, the individual inhibitory potencies of phenol, guaiacol, furfural, levulinic acid, HMF and vanillin (2.0 g/L) on the fermentation of glucose at a high concentration were evaluated by comparing the results with that of the control in batch fermentation. Sugar solution without an inhibitor was used as control. The results in Supplementary Figure S1 show that phenol and guaiacol severely inhibited ethanol production during the 72 h fermentation period at the concentration of 2 g/L or higher^32^,^33^. It is noted that phenol, guaiacol, vanillin, levulinic acid, furfural, and HMF each at 2.0 g/L decreased the concentration of produced ethanol by 59, 41, 13, 0, 15 and 8 g/L, respectively. The higher toxicity of phenol and guaiacol than that of furfural, HMF, vanillin or levulinic acid to the yeast in the fermentation is consistent with the literature^12^.

### Inhibition of phenol on the ethanol production from glucose

**Figure 1:**
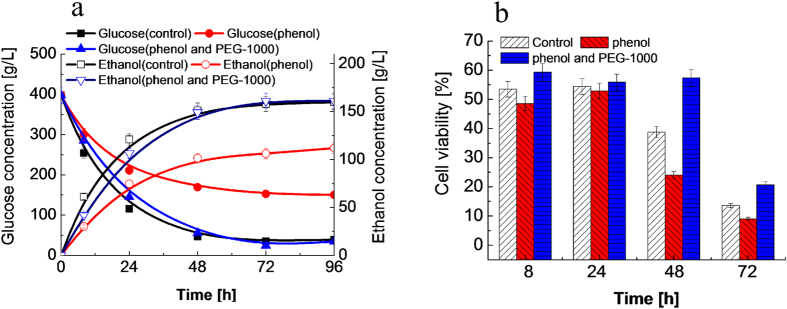
Inhibition of phenol on ethanol production from glucose. (**a**) Profiles of glucose consumption and ethanol productivity during glucose fermentation process. (**b**) Yeast cell viability from glucose fermentation processes. Fermentation conditions: 398 g/L glucose, 2.0 g/L of phenol, approximately 5.0 × 10^8^ cells/mL, 0.25 g/mL of PEG-1000, 33 ^o^C, 160 rpm, and pH of 4.3.

Results in Supplementary Figure S1 show that phenol has the strongest inhibition on ethanol production. Therefore, we first investigated the inhibition of phenol on the ethanol production from glucose in great details. Figure 1a shows the ethanol and glucose concentration profiles observed during glucose fermentation. Phenol drastically inhibited ethanol productivity and glucose consumption during the 96 h fermentation period compared to the control, producing 111 g/L of ethanol as compared to 158 g/L of ethanol in the control. However, when the same fermentation broth was added with 0.25 g/mL of PEG-1000, the ethanol productivity and glucose consumption were pronouncedly improved and became comparable to that without phenol inhibitor (control) after 48 h. The results indicate that the VHG fermentation in the presence of inhibiting phenol was much improved by PEG-1000.

Based on the data presented in Fig. 1a, we hypothesized that yeast cell viability is critically correlated with the ethanol yield. Therefore, we measured^34^ the yeast cell viabilities of the different compositions as shown in Fig. 1b. The presence of 2.0 g/L phenol resulted in only 24% yeast cell viability, much lower than the 39% viability for the control after 48 h fermentation period. When the same fermentation broth was added 0.25 g/mL of PEG-1000, the yeast cell viability was remarkably increased to 58%, even higher than that of the control, after 48 h. In addition to detoxifying phenol in the lignocellulosic hydrolysate, PEGs also protected the industrial dry yeast (starch-base) cells under high ethanol concentration. As reported in the VHG fermentation^31^, the vitality of the yeast cells and their tolerance to high ethanol titer were boosted when PEG-1000 was present in the fermentation broth.

### The effect of PEG-1000 on the ethanol production at different phenol concentrations

**Figure 2:**
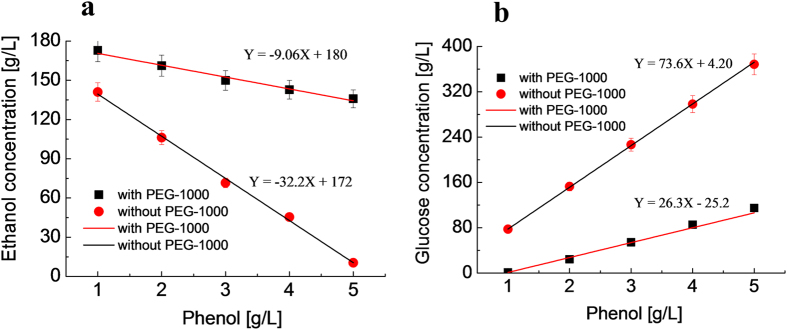
The effect of PEG-1000 during fermentation at different phenol concentrations. (**a**) Correlation between ethanol concentration and phenol concentration. (**b**) Correlation between glucose concentration and phenol concentration. Fermentation conditions: 398 g/L glucose, approximately 5.0 × 10^8^ cells/mL, 0.25 g/mL of PEG-1000, 33 ^o^C, 72 h, 160 rpm, and pH of 4.3.

To determine the detoxification efficacy of PEG-1000, the inhibitory effect of different phenol concentration (between 0–5.0 g/L) on the ethanol production from glucose was further quantitatively determined for 72 h fermentation (Supplementary Fig. S3). Higher concentration of phenol (5.0 g/L) resulted in almost complete inhibition of ethanol production; the produced ethanol only reached a marginal concentration of 10 g/L as compared to 158 g/L for the control. However, when 0.25 g/mL of PEG-1000 was added to the same fermentation broth, the ethanol concentration was dramatically increased from 10 g/L to 135 g/L, with 114 g/L of glucose unconverted in the fermentation broth. While a low concentration of phenol (1.0 g/L) also inhibited ethanol production (141 g/L of ethanol), PEG-1000 addition increased the ethanol concentration to 172 g/L with only 1.0 g/L of unconverted glucose. A linear correlation was observed between phenol concentration (x) and ethanol concentration (y) at phenol concentration range of 1.0–5.0 g/L (Fig. 2a) with or without PEG-1000. The ethanol concentration decreases with increasing phenol concentration. The different slopes, −32.2 without PEG-1000 and −9.06 with PEG-1000 indicate that the inhibition of phenol to ethanol production was significantly alleviated by the presence of PEG-1000. The inversely proportional relationship between phenol and unconverted glucose concentration illustrated in Fig. 2b also shows the beneficial effect of the supplementation of PEG-1000 that improved glucose conversion (Supplementary Fig. S3) during glucose fermentation. However, glucose in the medium was increased at high phenol level. One solution to this problem is to optimize the ratio of PEGs loading to the inhibitor’s concentration. It should be noted that this part of study is based on an artificially high concentration of phenol model compound. In some real biomass hydrolysates, as in the pretreated poplar sample reported in this work below, the phenol concentration could be considerably lower.

### The effect of PEG-1000 during fermentation in the presence of mixed inhibitors

**Figure 3:**
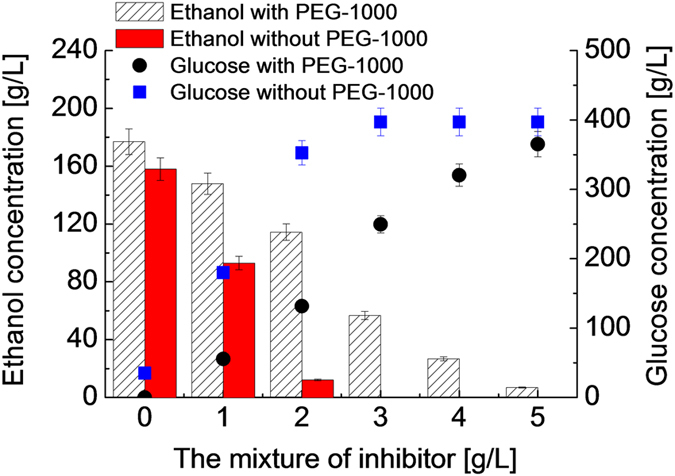
The effect of PEG-1000 on glucose fermentation at different concentrations of mixed inhibitors. Fermentation conditions: 398 g/L glucose, approximately 5.0 × 10^8^ cells/mL, 0.25 g/mL of PEG-1000, 33 °C, 72 h, 160 rpm, and pH of 4.3.

The speciation and concentration of inhibitors may vary depending on the types of biomass and also on pretreatment processes and conditions. Different inhibiting molecules have been shown to reduce yeast cell growth and ethanol production^35^. To determine the reciprocal effect of the mixed inhibitors, the inhibitory effect of a combination of six inhibitors (phenol, guaiacol, vanillin, levulinic acid, furfural and HMF) on ethanol production from glucose was evaluated (Fig. 3). The fermentation activity of the yeast was inhibited with increasing inhibitor’s concentrations, as evidenced by the reduced glucose conversion and the ethanol productivity expressed in concentration (Fig. 3 and Supplementary Fig. S4).

Another important observation is that the decrease in glucose conversion and ethanol productivity due to the mixed inhibitors are not linearly dependent on the inhibitor concentration (Supplementary Fig. S4). The ethanol concentration produced in the presence of 0, 1.0 and 2.0 g/L each of the mixed inhibitors are 158, 93, and 12 g/L, respectively (Fig. 3). Evidently, the higher inhibitor concentration resulted in more pronounced decrease in the ethanol productivity. The decrease in glucose conversion follows the same trend. Importantly, the combination of the six inhibitors (2 g/L each) showed reciprocal inhibition on ethanol production, by causing 146 g/L decrease in the ethanol concentration, compared to the decrease of 128 g/L expected from the sum of the individual compounds (Supplementary Fig. S1). This observation may be ascribed to the inhibitor reciprocal effect. The reciprocal effect and the loading effect of the mixed inhibitors, typically exist in lignocellulosic hydrolysate, can be expected to cause severe inhibition to the yeast fermentation performance.

When 0.25 g/mL of PEG-1000 was added the same fermentation broth (Fig. 3), the ethanol concentration was increased from 93 g/L to 147 g/L under 1.0 g/L each of the inhibitors. The robustness of the fermentation system was further demonstrated by the production of ethanol even when the inhibitor concentration was increased up to 5.0 g/L each; the fermentability was improved under *in-situ* detoxification by PEG-1000.

### The effect of PEG-1000 during ethanol production from low glucose concentration in the presence of mixed inhibitors

**Figure 4:**
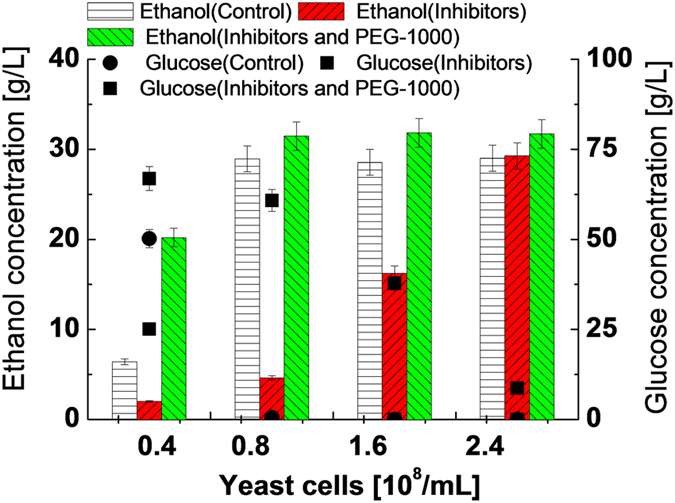
The effect of PEG-1000 during ethanol production from low glucose concentration in the presence of mixed inhibitors. 72 g/L glucose, 2.0 g/L each of mixed inhibitors, 0.2 g/mL of PEG-1000, 33 °C, 48 h, 160 rpm, and pH of 4.3.

In light of the strikingly positive results achieved from the supplementation of PEG-1000 in the fermentation broth, we moved to verify this chemical *in-situ* detoxification strategy for the fermentation of a lignocellulosic hydrolysate to produce ethanol. It should be noted that VHG fermentation process is not yet directly applicable to the hydrolysate derived from lignocellulose due to pretreatment limitations to produce high sugar concentration from a whole biomass. In real lignocellulosic hydrolysate, the glucose concentration would be much lower than that used in high gravity fermentation. We therefore further evaluated simulated lower glucose concentration to establish the baseline of ethanol production in the presence of 2.0 g/L of mixed inhibitors. Fermentation was evidently inhibited when the yeast cell concentration is lower than 1.6 × 10^8^/ml (Fig. 4 and Supplementary Fig. S5). The ethanol and glucose concentrations became comparable with the control (Fig. 4), even at the yeast cell concentration lower than 0.8 × 10^7^/mL due to the supplementation of PEG-1000 in the fermentation broth. The presence of PEG-1000 again resulted in dramatically enhanced ethanol productivity. The inhibition by 2.0 g/L of inhibitors was nearly fully lifted by increasing the yeast cell concentration to 2.4 × 10^8^ /mL.

Effect of different PEGs in molecular weight on batch ethanol fermentation was investigated. Five PEGs were evaluated in this work (Supplementary Fig. S6). When PEGs molecular weight is higher than 400, the glucose conversion, ethanol yield and concentration reached a maximum. In this study, PEG-1000 was chosen and studied in greater detail as the appropriate additive in improving fermentation productivity.

We also investigated the relationship between PEG concentration and the fermentation efficiency in glucose fermentation process with 2.0 g/L reach of mixed inhibitors. The ethanol concentration increases gradually with the increase in PEG-1000 concentration. As the concentration of PEG-1000 reached 0.20 g/mL, 32.0 g/L of ethanol (86% of ethanol yield) was obtained and no residual sugars remained in the fermentation broth (Supplementary Fig. S7). These results served as the basis for an appropriate PEGs concentration.

### Integrated recycle process for yeast, PEGs and H_2_SO_4_

In order to improve the process economics and reduce wastewater disposal, we further studied the separation of inhibitors, and the recovery and reuse of the PEGs. An integrated recycle process for yeast, PEG-1000 and H_2_SO_4_ was developed (Supplementary Fig. S8). In the experiments, fresh yeast cells were supplemented in late cycles for complete glucose-to-ethanol conversion. The yeast cells were first recovered by centrifugation. The PEG-1000 and H_2_SO_4_ were recycled after removing ethanol and water by distillation and removing inhibitors by extraction from the crude PEG mixture. The living cells and PEG-1000 were recycled for use in subsequent fermentation process. Fresh yeast cells were supplemented in cycles 1–3 with the amount pre-determined based on the death rate of cells (55%, 77%, 77%, respectivily) according to the results in Supplementary Fig. S9. There was no obvious change in glucose conversion, ethanol yield and concentration from the four repeated uses of the initially loaded PEG-1000 and H_2_SO_4_.

### Simultaneous saccharfication and co-fermentation (SSCF) of H&E-poplar

In order to confirm the potential *in-situ* detoxification by PEGs for the conversion of a real world feedstock, we investigated the conversion of a mildly pretreated lignocellulose by combining enzymatic saccharification and simultaneous co-fermentation (SSCF) using a starch-base industrial *S. cerevisiae* yeast. As a firm demonstration, ethanol production from a poplar after steam hydrolysis and explosion (H&E) was carried out through the SSCF process (Supplementary Fig. S10). It should be noted that the steam hydrolysis step was also intended to extract xylose sugar from hemicellulose under a mild condition which was in sufficient quality to produce furfural. This hydrolysis resulted in the efficient removal of xylan as indicated by Table 1. While the concentrations of phenol, HMF, and acetic acid are low in the H&E poplar, the lignin and cellulose contents are preserved from the pretreatment, showing as an increased content due to the removal of hemicellulose (Table 1).

**Figure 5:**
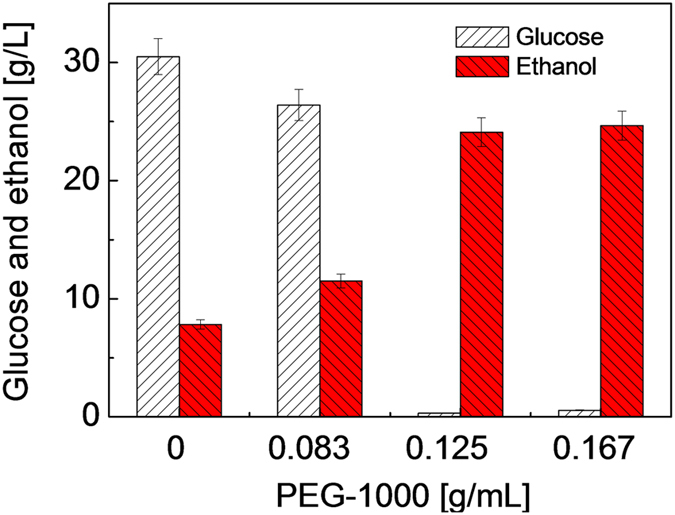
The simultaneous saccharification and co-fermentation (SSCF) of H&E-poplar. Conditions: 33 °C, 24 h pre-hydrolysis at pH 4.8 and 12.5% DW loading (see experimental), 30FPU/g dry DW, approximately 0.8 × 10^8^ cells/mL, 72 h, 160 rpm.

It was observed that the glucose concentration from the enzymatic hydrolysis was sufficiently high from the H&E poplar (Fig. 5). However, while the unconverted glucose concentration was at 30.5 g/L, only 7.0 g/L of ethanol was obtained without PEGs. Remarkably, increasing amount of glucose was converted to ethanol with increased PEG-1000 loading in the SSCF system. A most notable change occurred at PEG-1000 loading of 0.125 g/mL. It is apparent that enzymatic hydrolysis of the H&E poplar to glucose was not a rate limiting step for the production of ethanol. The fermentation appeared inhibited by the presence of lignin as evidenced by the high unconverted glucose concentration (30.5 g/ L). The results indicate that the lignin toxicity prevails in the fermentation step of the SSCF process. Addition of PEG-1000 evidently alleviated the lignin toxicity in fermentation. A pH of 4.8 is used in this SSCF process as it is most suited for the enzymatic saccharification and is within the optimal performance range of the yeast. The ethanol concentration increases and glucose concentration decreases concurrently as the PEG-1000 concentration increase. A maximum ethanol concentration of 24.0 g/L was achieved with only 0.4 g/L of glucose remained. In the presence of PEG-1000, ethanol productivity was enhanced by 3 fold as compared to that in the absence of PEGs in the fermentation broth. The results therefore demonstrate that it is feasible to directly process a real world lignocellulose through enzymatic saccharification and simultaneous co-fermentation without excessive detoxification steps prior to fermentation in the presence of the fully water soluble PEGs.

### Mechanistic study on the role of PEGs for *in-situ* detoxification

**Figure 6:**
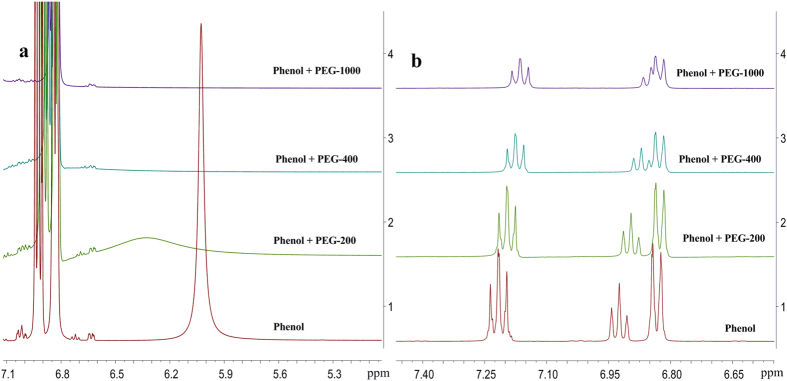
^1^H NMR spectra of phenol in PEGs. (**a**) ^1^H NMR spectra of the hydroxyl group of phenol, with and without PEGs, in CDCl_3_. (**b**) Partial ^1^H NMR spectra of phenyl ring, with and without PEGs, in CDCl_3_.

One hypothesis on the *in-situ* detoxification mechanism was the *in situ* adsorption of inhibitor molecules by PEGs. The PEG molecules could form a protective shell around the surface of the cells. In this hypothesis, the PEGs may shield the yeast cells from being attacked by the inhibitor molecules. Therefore, the interaction of PEGs with phenol, the most potent inhibitor molecule, was investigated by ^1^H NMR spectroscopic measurement of phenol dissolved in PEGs of different chain lengths, using that of phenol in CHCl_3_ as a reference (Fig. 6). It was found that the chemical shift of proton in phenol hydroxyl group moved to the low field, and proton peak broadened and disappeared as the molecular weight of PEGs was increased. Conversely, the chemical shifts of the three types of protons in the phenyl ring moved to the high field. The NMR results clearly indicate that there is a strong hydrogen bonding interaction of the proton of phenol hydroxyl group with PEGs, most likely with the ether oxygens. Experimentally, we found PEG-200 was a less effective detoxification agent compared to PEG-400 or higher. Remarkably, the broadened ^1^H NMR peak is consistent with the fact that PEG-200 does not effectively shield the phenolic protons from the yeast cells. Furthermore, the ^1^H NMR results of PEG-400 and PEG-1000 support our hypothesis for a strong interaction between the higher PEGs and phenolic protons, resulting in the increased yeast cells tolerance and ethanol productivity, in good agreement with the above fermentation results (Supplementary Fig. S6). The formation of the hydrogen bonding reduces the electron cloud density around the nucleus of H atom of phenol hydroxyl group; the chemical shift was significantly increased due to the de-shielding effect. Due to the new hydrogen bonding formation with the oxygens in the PEGs, the hydroxyl H-O bonding in the phenol becomes weaker, leading to an increase in the electron cloud density of phenyl ring, and consequently the up-field chemical shift of the phenyl ring protons.

## Discussion

Cellulosic ethanol is a sustainable renewable bio-fuel because it helps greatly reduce the net greenhouse gas emissions. To enable an industrial process for ethanol production, various methods have been extensively studied that mainly focused on removing inhibitors from fermentation broth and on improving tolerance of microorganisms to the toxic inhibitors^13^,^14^,^15^.

Several strategies on detoxification of lignocellulosic hydrolysates have been reported. A recent study showed that a non-toxic surfactant (L62D and L62LF) based cloud point extraction (CPE) two-phase system preferentially extracted over 90% of phenolic compounds, and less than 20% of acetic acid and HMF inhibitors from a model hydrolysate^36^. This and other reported methods carry limitations including specific affinities of detoxifying agent, sugar loss, and additional filtration steps^16^,^17^,^18^,^19^,^20^,^21^,^22^.

As described in the Introduction, the use of recombinant yeast strains improves fermentability of lignocellulosic hydrolysate and avoids loss of sugars. However, longer incubation times and complex incubation process of recombinant yeast strains increase production costs and biological risk^37^. The adaptation of the laboratory strain to the very harsh industrial fermentation conditions has not been reported. The fermentation processes under harsh industrial environmental conditions, especially using lignocellulosic hydrolysates, require strains that are robust, and have the ability to adjust rapidly their metabolism to adapt to a specific environment over a long time^38^. Saccharomyces cerevisiae industrial strain has been widely used in commercial fermentation of ethanol from sugars and starch sources^39^, but this yeast has not been adapted to fermentation in the presence of toxic compounds derived from the lignocellulose pretreatment process.

In this study, we successfully demonstrated the feasibility of (1) fermenting high gravity sugars in the presence of high concentrations of toxic inhibitors, and (2) directly fermenting a lignocellulosic hydrolysate derived from mildly pretreated biomass by industrial yeast (starch-base) cells without extra detoxication steps. By PEG *in-situ* detoxification, high fermentation efficiency and ethanol productivity are achieved. The PEGs are shown to effectively improve the yeast cell performance to produce ethanol in high productivity in the presence of known toxic inhibitory compounds, including lignin. Importantly, an industrial *S. Cerevisiae*, which has been widely used for ethanol production from starch sugars in commercial processes, showed robust performance enabled by the presence of PEGs. The potential economic benefits of PEGs can be achieved by the elimination of additional steps to detoxify the hydrolysate after mild pretreatment with minimal loss of carbohydrates.

PEGs and yeast cell loss can have a significant impact on the economics. Therefore, an integrated recycle process for yeast, PEG-1000 and H_2_SO_4_ was designed and successfully demonstrated in this work. The recovered living cells and PEG-1000 with H_2_SO_4_ were reusable for the subsequent fermentations. The above results (Supplementary Fig. S6) show that PEGs in different molecular weights have similar performance. Therefore, PEG-1000 was chosen to study recycle process detail for yeast, PEGs and H_2_SO_4_. Overall, in addition to efficient fermentation performance of the industrially available yeast under *in-situ* the detoxification by PEGs, the overall cost for the production of cellulosic ethanol can also be further reduced due to the technical feasibility of recycling the living yeast cells and the chemicals.

The yeast cell viability is an important measure of the robustness of ethanol-producing yeasts. Figure 1b shows the dramatic difference in yeast cell viabilities in the presence and absence of PEG-1000. The relatively high yeast cell viability is one of the factors responsible for the high ethanol productivity due to the presence of PEG-1000 (Fig. 1a). Based on our previous work, PEG-400 can provide protection to the yeast cells and improve their vitality under VHG conditions, resulst in increasing ethanol productivity^31^. It is particularly important to note that PEGs significantly increased ethanol productivity even in the presence of a high concentration of phenol under VHG conditions. PEGs not only protect yeast cells to enhance their vitality under VHG conditions, but also exhibits *in situ* detoxifition to the inhibitor (phenol) in the lignocellulosic hydrolysate. The ^1^H NMR spectroscopic results (Fig. 6) provide clear evidences for the chemical interaction of PEGs with inhibitors such as phenol, which is consistant with our hypothesis on the role of the PEGs in the fermentation broth. This point will be discussed in greater detail later.

Because the cellulase catalyzed hydrolysis of cellulose to produce fermentable sugars is also inhibited by glucose and cellobiose in the hydrolysate, the consumption of glucose by immediate fermentation to ethanol may be expected to alleviate this problem. Therefore, simultaneous saccharification and co-fermentation process was suggested for cellulosic ethanol production in which the cellulase and yeast co-exist and complement in the overall process^40^. While some PEGs have been used in the enzymatic hydrolysis of cellulose from lignocellulosic mass to improve the enzymatic efficiency^41^, we further demonstrated the potential of PEG chemically exo-protected industrial starch-base *S. Cerevisiae* for ethanol production throuth a simultaneous saccharification and co-fermentation process from a representative real world lignocellulose feedstock. For a polar pretreated by low-cost steam hydrolysis combined with explosion, PEG-1000 enhanced ethanol productivity by 3 fold as compared to that in the absence of PEGs in the fermentation broth. Compared with all prior studies, our work demonstrated significant advantages by eliminating detoxification steps of pretreated solids. It is reasonable to expect that ethanol concentration and the bioconversion productivity could be improved by optimizing SSCF condition, and the related investigation is now in progress.

The strong interaction of PEGs with phenol as revealed by the NMR study of this work (Fig. 6) provides some preliminary insight to the mechanism of exo-protection by the PEGs. In the literature, inhibition mechanisms of phenolic compounds on *S. cerevisiae* have not yet been completely elucidated^42^. It was suggested that phenolic compounds may act on biological membranes, causing loss of integrity, thereby affecting their ability to serve as selective barriers and enzyme matrices^43^. Weakly acidic phenolic compounds may destroy the electrochemical gradient by transporting the protons back across the mitochondrial membranes^44^. Base on the detoxification fermentation and the NMR spectroscopic results of this work, a possible mechanism of exo-protection by PEGs to the yeast cells is proposed. The stronger hydrogen bonding interaction of phenol hydroxyl group with PEGs prevent phenolic compounds from acting on biological membranes, and transporting the protons back across the mitochondrial membranes. As a result, the cell integrity was maintained.

**Figure 7:**
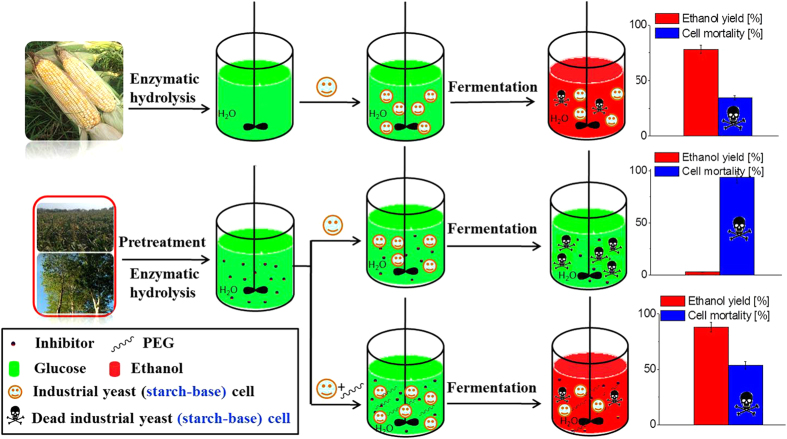
Starch-base industrial *S. cerevisiae* yeast, under *in-situ* detoxification by PEGs in fermentation broth, displayed unabated ethanol productivity from cellulosic hydrolysate.

In summary, we demonstrated the feasibility of chemically exo-protected starch-base industrial *S. cerevisiae* yeast cells by PEGs to convert lignocellulose mass to ethanol. The essential role of PEGs in enabling the fermentation of cellulosic glucose is illustrated in Fig. 7. The industrial *S. cerevisiae* yeast (starch base) directly converted the glucose from hydrolysate of pretreated lignocelluloses to ethanol through *in-situ* detoxification of PEGs. Furthermore, the industrial *S*. *cerevisiae* yeast cells (starch base) can be directly used without the need for genetically modified microorganisms in a large scale industrial process. The surviving yeast cells and PEGs can be recycled with sustained fermentation performance. The *in-situ* detoxification achieved by PEG adsorption of inhibitors in the fermentation broth enhanced ethanol production. This detoxification approach may be a useful tool for biofuel production from lignocellulosic hydrolysates that contain toxic compounds. Future research will focus on optimizing the biomass pretreatment process in conjunction with the integrated detox fermentation process for optimized ethanol yield, and on fully establishing the economic feasibility of this technology in large scales.

## Meterials and Methods

### Organisms and chemicals

The microorganism used for fermentationwas Saccharomyces cerevisiae in the form of dry yeast (thermal resistant) (Angel Yeast Company Ltd, Yichang, China), which was named Angel Super-Alcohol Active Dry Yeast (starch base). The yeast was kept at 4 ^o^C during storage, and was weighted and directly added to specified fermentation media as received right before each use. Filter paper activity of cellulase (CTec-2) was 150 FPU/mL, kindly provided by Novozymes Investment Co. Ltd. (Beijing, China). D-glucose (C_6_H_12_O_6_·H_2_O), glycerol (99 wt%), ethanol (99 wt%) were received from Sinopharm (China). Phenol, guaiacol, vanillin, levulinic acid, furfural and 5-hydrolsymethylfurfural (HMF), were received from Sinopharm (China). Polyethylene glycol were purchased from Aladdin(China). Sulphuric acid (98wt%) and methylene blue were provided by a local supplier. Deionized water (DI H_2_O) was produced by a Milli-Q Integral 5 system. All other chemicals were of analytical quality.

### Measurement of viable cell density

The yeast viability was measured according to the Methylene-Violet Staining procedure^34^. A volume of 100 μl dilute sample containing cells was mixed with 100 μl of a methylene blue solution. After 20 min staining, the numbers of viable (living) cells and of total cells were counted under a microscope (Nikon Ci-L). The cell viability and mortality are calculated according to the following equation:


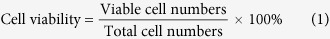



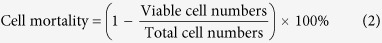


###  Detox fermentation of glucose

Fermentation broths were prepared in 100 or 250 mL flasks with 10–50 mL of 70–400 g/L glucose using ultrapure water. To study the effect of fermentation inhibitors, we added to the fermentation medium each of six well-known inhibitors typical of cellulosic ethanol fermentation: phenol, guaiacol, vanillin, levulinic acid, furfural and 5-hydromethylfurfural (5-HMF) in the range of 0–6 g/L. Different amounts (0.005–0.25 g/ml) of PEG-1000 were added to the fermentation media. Commercially available immobilized active dry yeast was added to fermentation broth directly without additional incubation step, the initial yeast cell concentration was approximately 0.8 × 10^8^–5 × 10^8^ cells/mL. No additional nutrients were applied during fermentation. The pH of all fermentation containing inhibitors was adjusted to 4.3 using H_2_SO_4_ solution. For reference, sugar solutions with no inhibitor and surfactant were used as controls. Since the objective of this part of the study was to evaluate the fermentability of the lignocellulosic hydrolysate and the effectiveness of the hypothesized detoxification method, glucose was used as the sole carbon source and the fermentation did not include 5-carbon sugars and nutrient. The fermentation was performed in batch mode and the temperature was controlled at 33 °C. During fermentation, the flasks were placed on a rotary shaker (ZWY-240) at 160 rpm. All the experiments were conducted in duplicate with the average and standard deviation shown in figures.

### Procedure for the recovery and reuse of yeast, PEGs and H_2_SO_4_

A representative procedure for the recovery and reuse of yeast, PEGs and H_2_SO_4_ is shown in Supplementary Fig. S8. After fermentation, the yeast cells were collected by centrifugation at 8000 rpm for 5 minutes, and the yeast cells were further evaluated for their activity in subsequently fermentations. The separated liquid phase was subjected to vacuum distillation to separate ethanol and water, and the residual mixture was then subjected to extraction using ether to remove inhibitors, the residual mixture was distillated to obtained PEGs with H_2_SO_4_. Recovered yeast and PEGs with H_2_SO_4_ were added to the subsequently fermentation process together.

### Hydrolysis combined with steam explosion (H&E) pretreatment of poplar

Poplar chips obtained from Shandong province of China (L/W/H ≈ 30 mm/20 mm/4 mm). The poplar was pretreated to decompose hemicellulose into furfural and to fragment the poplar chips to small particles. H&E pretreatment was conducted in a pressure-tight stainless steel reactor under the following conditions: 150 g poplar mixed with 150 g deionized water was loaded into the hydrolysis reactor (I.D 48 mm * 500 mm). A saturated steam at 205–210 °C was injected into the reactor continually until the temperature reached target value (205–210 °C). The hydrolysis condition was maintained at this temperature for 30 min by supplementing steam as needed. The valve to the explosion vessel was then quickly opened to allow all the materials to be pushed into a collecting container. The collected wet H&E pretreated poplar was dried at room temperature under ventilation until the moisture was less than 10%. The composition of the solid fraction is named H&E poplar. H&E poplar was analyzed for chemical composition following the procedure of the U.S. National Renewable Energy Laboratory^45^. A two stage acid hydrolysis procedure was used for analyze the chemical compositions of the H&E poplar solids. Klason lignin was measured gravimetrically. The compositions of untreated and pretreated poplar were listed Table 1.

### Detox SSCF of H&E-poplar

The H&E-poplar was used as a substrate for simultaneous saccharification and co-fermentation (SSCF) (Supplementary Fig. S8). The SSCF experiments were carried out in duplicates using shaking flasks in an orbital incubator at 160 rpm. The initial pH of the medium was adjusted to 4.8 using sodium acetate buffer solution. 12.5% dry matter (DW) loading was used. PEG-1000 of 0.05–0.2 g/mL and cellulase (30FPU/g DW) were added to the poplar slurry. The enzymatic hydrolysis of 24 h at 50 °C followed by changing conditions to 33 °C, and inoculating yeast cells approximately 0.8 × 10^8^ cells/mL. After 72 h of SSCF, the resultant slurry was centrifuged to remove the solid material and the supernatant was diluted, and analyzed by HPLC using a 25ul injection volume. The ethanol yield was defined as the amount of ethanol after 72 h incubation on the basis of theoretical amount of ethanol in the pretreated substrate. The ethanol concentration was defined as the amount of ethanol after 72 h incubation on the basis of buffer volume. All the experiments were conducted in duplicate with the average and standard deviation shown in figures.

### HPLC analysis

The sample of each fermentation broth was diluted with deionized water, and filtered through a 0.22um filter. The glucose and ethanol concentrations of fermentation samples were quantified using a high performance liquid chromatography (HPLC). An Agilent 1260 Series HPLC system equipped with a refractive index detector was used. Ion exchange columns (HPX-87H, 300 × 7.7 mm) were used in series. The column and detector temperatures were maintained at 65 ^o^C and 50^o^C, respectively, with 5 mM H_2_SO_4_ as the mobile phase at 0.6mL/min. The glucose conversion was calculated based on initial glucose and consumed glucose. The ethanol yield was calculated based on the theoretical ethanol yield from consumed glucose; the concentrations of ethanol and glucose were calculated based on the water volume in the fermentation broth. Data analyses were performed using the Agilent Chemstation software and Microsoft Excel. The glucose conversion, ethanol yield and ethanol concentration were calculated according to the following equations:


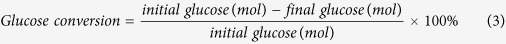



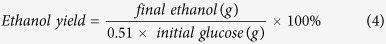



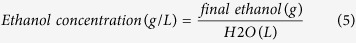



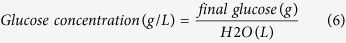


### NMR spectroscopy analysis

NMR spectra were measured in CDCl_3_ on a 400 MHz instrument and recorded at the following frequencies: proton (^1^H, 400 MHz), carbon (^13^C, 100 MHz). ^1^H NMR chemical shifts were reported in ppm using tetramethylsilane (TMS, δ (ppm) = 0.00 ppm) as the internal standard. ^13^C NMR spectra were reported in ppm using CDCl_3_ as the internal standard.

## Additional Information

**How to cite this article**: Liu, X. *et al.* Lignocellulosic ethanol production by starch-base industrial yeast under PEG detoxification. *Sci. Rep.* **6**, 20361; doi: 10.1038/srep20361 (2016).

## Supplementary Material

Supplementary Information

## Figures and Tables

**Table 1:** The compositions of untreated and H&E pretreated poplar.

	UT-poplar^a^	H&E-poplar^b^
Glucan /%	42.34	50.40
Xylan /%	15.23	2.73
Lignin/%	25.40	34.72
Ash/%	0.47	0.48
HMF/%	nd	0.23
Acetic acid/%	nd	1.08
Phenol / %	nd	0.01

^a^UT-Poplar: untreated poplar.

^b^H&E-poplar: steam hydrolyzed and exploded poplar.

^c^nd: not detected.

## References

[b1] Hendriks A. T. W. M. & Zeeman G. Pretreatments to enhance the digestibility of lignocellulosic biomass. Bioresour. Technol. 100, 10–8 (2009). 18599291 10.1016/j.biortech.2008.05.027

[b2] Wei N., Quarterman J., Kim S. R., Cate J. H. D. & Jin Y. S. Enhanced biofuel production through coupled acetic acid and xylose consumption by engineered yeast. Nat. commun. 4, 1–7 (2013). 10.1038/ncomms3580 24105024

[b3] Farrell A. E. *et al.* Ethanol can contribute to energy and environmental goals. Science 311, 506–508 (2006). 16439656 10.1126/science.1121416

[b4] Koppram R., Tomas-Pejo E., Xiros C. & Olsson L. Lignocellulosic ethanol pr**o**duction at high-gravity: challenges and perspectives. Trends Biotechnol. 32, 46–53 (2014). 24231155 10.1016/j.tibtech.2013.10.003

[b5] Bellido C. *et al.* Effect of inhibitors formed during wheat straw pretreatment on ethanol fermentation by Pichia stipitis. Bioresour. Technol. 102, 10868–10873 (2011). 21983414 10.1016/j.biortech.2011.08.128

[b6] Ludwig D., Amann M., Hirth T., Rupp S. & Zibek S. Development and optimization of single and combined detoxification processes to improve the fermentability of lignocellulose hydrolyzates. Bioresour. Technol. 133, 455–461 (2013). 23454802 10.1016/j.biortech.2013.01.053

[b7] Cheng J. L., Leu S. Y., Zhu J. Y. & Jeffries T. W. Ethanol production from non-detoxified whole slurry of sulfite-pretreated empty fruit bunches at a low cellulase loading. Bioresour. Technol. 164, 331–337 (2014). 24874873 10.1016/j.biortech.2014.04.102

[b8] Jorgensen H., Vibe-Pedersen J., Larsen J. & Felby C. Liquefaction of lignocelluloses at high-so*lids concentratio*ns. Biotechnol. Bioeng. 96, 862–870 (2007). 16865734 10.1002/bit.21115

[b9] Felby C., Thygesen L. G., Kristensen J. B., Jorgensen H. & Elder T. Cellulose-water interactions during enzymatic hydrolysis as studied by time domain NMR. Cellulose 15, 703–710 (2008).

[b10] Jönsson L. J., Alriksson B. & Nilvebrant N. O. Bioconversion of lignocellulose: inhibitors and detoxification, Biotechnol. Biofuels 6, 16 (2013). 23356676 10.1186/1754-6834-6-16 PMC3574029

[b11] Liu Z. L. Genomic adaptation of ethanologenic yeast to biomass conversion inhibitors. Appl. Microbiol. Biotechnol. 73, 27–36 (2006). 17028874 10.1007/s00253-006-0567-3

[b12] Palmqvist E. & Hahn-Hagerdal B. Fermentation of lignocellulosic hydrolysates. II: inhibitors and mechanisms of inhibition. Bioresour. Technol. 74, 25–33 (2000).

[b13] Helle S., Cameron D., Lam J., White B. & Duff S. Effect of inhibitory compounds found in biomass hydrolysates on growth and xylose fermentation by a genetically engineered strain of *S-cerevisiae*. Enzyme Microb. Technol. 33, 786–792 (2003).

[b14] Klinke H. B., Thomsen A. B. & Ahring B. K. Inhibition of ethanol-producing yeast and bacteria by degradation products produced during pre-treatment of biomass. Appl. Microbiol. Biotechnol. 66, 10–26 (2004). 15300416 10.1007/s00253-004-1642-2

[b15] Li H. Q. & Chen H. Z. Detoxification of steam-exploded corn straw produced by an industrial-scale reactor. Process Biochem. 43, 1447–1451 (2008).

[b16] Palmqvist E. & Hahn-Hagerdal B. Fermentation of lignocellulosic hydrolysate. I: inhibition and detoxification. Bioresour. Technol. 74, 17–24 (2000).

[b17] Nigam J. N. Development of xylose-fermenting yeast *Pichiastipitis* for ethanol production through adaptation on hardwood hemicelluloses acid prehydrolysate. J. Appl. Microbiol. 90, 208–215 (2001). 11168723 10.1046/j.1365-2672.2001.01234.x

[b18] Berson R. E., Young J. S., Kamer S. N. & Hanley T. R. Detoxification of actual pretreated corn stover hydrolysate using activated carbon powder. Appl. Biochem. Biotechnol. 121, 923–934 (2005). 15930571 10.1385/abab:124:1-3:0923

[b19] Keating J. D., Panganiban C. & Mansfield S. D. Tolerance and adaptation of ethanologenic yeasts to lignocellulosic inhibitory compounds. Biotechnol. Bioeng. 93, 1196–1206 (2006). 16470880 10.1002/bit.20838

[b20] Martin C. & Johnsson L. J. Comparison of the resistance of industrial and laboratory strains of *Saccharomyces* and *Zygosaccharomyces* to lignocellulose-derived fermentation inhibitors. Enzyme Microb. Technol. 32, 386–395 (2003).

[b21] Okuda N., Soneura M., Ninomiya K., Katakura Y. & Shioya S. Biological detoxification of waste house wood hydrolysate using *Ubreibacillus thermos-sphaericus* for bioethanol production. J. Biosci. Bioeng. 106, 128–133 (2008). 18804054 10.1263/jbb.106.128

[b22] Nicholas N. N. *et al.* Fungal metabolism of fermentation inhibitors present in corn stover dilute acid hydrolysate. Enzyme Microb. Technol. 42, 624–630 (2008).

[b23] Almeida J. R. M., Bertilsson M., Gorwa-Grauslund M. F., Gorsich S. & Liden G. Metabolic effects of furaldehydes and impacts on biotechnological processes. Appl. Microbiol. Biotechnol. 82, 625–38 (2009). 19184597 10.1007/s00253-009-1875-1

[b24] Office of Science, and Office of Energy Efficiency and Renewable Energy. Breaking the Biological Barriers to Cellulosic Ethanol: A Joint Research Agenda. A Research Roadmap Resulting from the Biomass to Biofuels Workshop Sponsored by the U.S. Department of Energy. Biofuels Joint Roadmap. **June**, 118–154 (2006).

[b25] Hawkins G. M. & Doran-Peterson J. A strain of Saccharomyces cerevisiae evolved for fermentation of lignocellulosic biomass displays improved growth and fermentative ability in high solids concentrations and in the presence of inhibitory compounds. Biotechnol. Biofuels 4, 49 (2011). 22074982 10.1186/1754-6834-4-49 PMC3256112

[b26] Wallace-Salinas V. & Gorwa-Grauslund M. F. Adaptive evolution of an industrial strain of *Saccharomyces cerevisiae* for combined tolerance to inhibitors and temperature. Biotechnol. Biofuels 6, 151 (2013). 24139317 10.1186/1754-6834-6-151 PMC4015299

[b27] Alriksson B., Horváth I. S. & Jönsson L. J. Overexpression of *Saccharomyces cerevisiae* transcription factor and multidrug resistance genes conveys enhanced resistance to lignocellulose-derived fermentation inhibitors. Process Biochem. 45, 264–271 (2010).

[b28] Almario M. P., Reyes L. H. & Kao K. C. Evolutionary engineering of *Saccharomyces cerevisiae* for enhanced tolerance to hydrolysates of lignocellulosic bimass. Biotechnol. Bioeng. 110, 2616–2623 (2013). 23613173 10.1002/bit.24938

[b29] Cakar Z. P., Turanli-Yildiz B., Alkim C. & Yilmaz U. Evolutionary engineering of *Saccharomyces cerevisiae* for improved industrially important properties. Fems Yeast Res. 12, 171–182 (2012). 22136139 10.1111/j.1567-1364.2011.00775.x

[b30] Tamis W. L. M., van Dommelen A. & de Snoo G. R. Lack of transparency on environmental risks of genetically modified micro-organisms in industrial biotechnology. J. Clean. Prod. 17, 581–592 (2009).

[b31] Liu X. *et al.* Vitalized yeast with high ethanol productivity. RSC Adv. 4, 52299–52306 (2014).

[b32] Fenske J. J., Griffin D. A. & Penner M. H. Comparison of aromatic monomers in lignocellulosic biomass prehydrolysates. J. Ind. Microbiol. Biotechnol. 20, 364–368 (1998).

[b33] Jurado M., Prieto A., Martínez-Alcala Á., Martínez Á. T. & Martínez M. J. Laccase detoxification of steam-exploded wheat straw for second generation bioethanol. Bioresour. Technol. 100, 6378–6384 (2009). 19683434 10.1016/j.biortech.2009.07.049

[b34] Smart K. A., Chambers K. M., Lambert I., Jenkins C. & Smart C. A. Use of methylene violet staining procedures to determine yeast viability and vitality. J. Am. Soc. Brew. Chem. 57, 18−23 (1999).

[b35] Palmqvist E., Meinander Q., Grage H. & Hahn-H. & gerdal B. Main and interaction effects of acetic acid, furfural and phydroxybenzoic acid on growth and ethanol productivity of yeasts. Biotechnol. Bioeng. 63, 46−55 (1999). 10099580 10.1002/(sici)1097-0290(19990405)63:1<46::aid-bit5>3.0.co;2-j

[b36] Dhamole P. B., Wang B. & Feng H. Detoxification of corn stover hydrolysate using surfactant-based aqueous two phase system. J. Chem. Technol. Biotechnol. 88, 1744–1749 (2013).

[b37] Tanimura A. *et al.* Direct ethanol production from starch using a natural isolate, *Scheffersomyces shehatae*: toward consolidated bioprocessing. Sci. Rep. 5, 9593(2015). 25901788 10.1038/srep09593 PMC5386104

[b38] Pereira F. B., Guimaraes P. M. R., Teixeira J. A. & Domingues L. Selection of *Saccharomyces cerevisiae* strains for efficient very high gravity bio-ethanol fermentation processes. Biotechnol. Lett. 32, 1655–1661(2010). 20574836 10.1007/s10529-010-0330-9

[b39] Bai F. W., Anderson W. A. & Moo-Young M. Ethanol fermentation technologies from sugar and starch feedstocks. Biotechnol. Adv. 26, 89–105 (2008). 17964107 10.1016/j.biotechadv.2007.09.002

[b40] Sun Y. & Cheng J. Hydrolysis of lignocellulosic materials for ethanol production: a review. Bioresour. Technol. 83, 1–11 (2002). 12058826 10.1016/s0960-8524(01)00212-7

[b41] Zhang Y., Xu X., Zhang Y. & Li J. Effect of adding surfactant for transforming lignocellulose into fermentable sugars during biocatalysing, Biotechnol. Bioprocess Eng. 16, 930–936 (2011).

[b42] Almeida J. R. *et al.* Mini-Review Increased tolerance and conversion of inhibitors in lignocellulosic hydrolysates by *Saccharomyces cerevisiae*, J. Chem. Technol. Biotechnol. 82, 340–349 (2007).

[b43] Heipieper H. J., Weber F. J., Sikkema J., Keweloh H. & Debont J. A. M. Mechanisms of resistance of whole cells to toxic organic solvents. Trends Biotechnol. 12, 409–415 (1994).

[b44] Terada H. Uncouplers of oxidative phosphorylation. Environ. Health Perspect. 87, 213–218 (1990). 2176586 10.1289/ehp.9087213 PMC1567840

[b45] Sluiter A. *et al.* Determination of structural carbohydrates and lignin in biomass. USA: NREL Laboratory Analytical Procedure, 1–14 (2008).

