# Supplementary material for: Lignocellulosic ethanol production by starch-base industrial yeast under PEG detoxification
Source: Sci Rep. 2016 Feb 3;6:20361. doi: 10.1038/srep20361 (PMC4738253; doi:10.1038/srep20361)
Supplement: Supplementary Information [file srep20361-s1.pdf]

Lignocellulosic ethanol production by starch-base industrial yeast under PEG detoxification

Xiumei Liu, Wenjuan Xu, Liaoyuan Mao, Chao Zhang, Peifang Yan, Zhanwei Xu, and Z. Conrad Zhang*

State Key Laboratory of Catalysis, Dalian National Laboratory for Clean Energy, Dalian Institute of Chemical Physics, Chinese Academy of Sciences, Dalian 116023,P. R. China. E-mail:zczhang@yahoo.com.

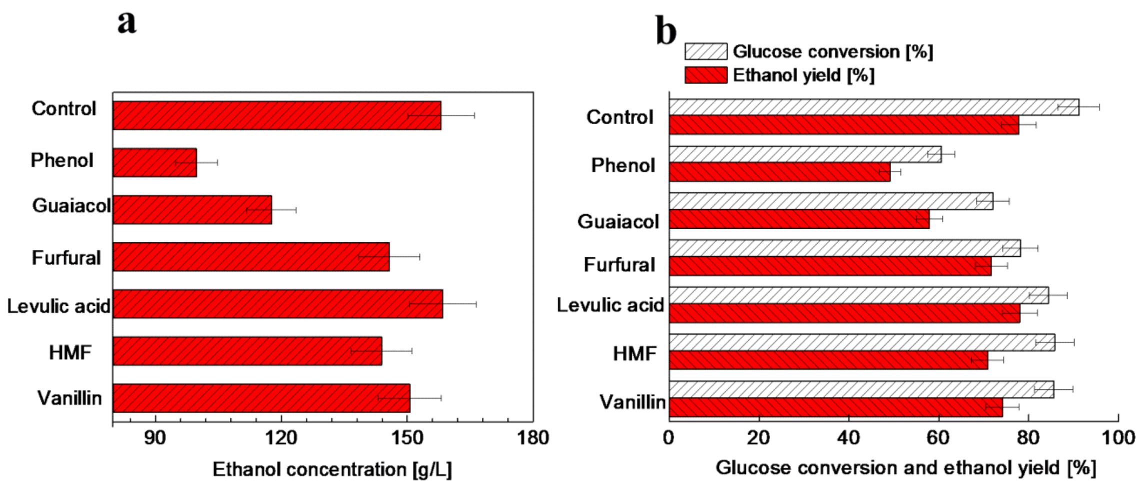

Supplementary Figure S1 Effects of phenol, guaiacol, furfural, levulinic acid, HMF and vanillin on ethanol productivity in reference to the control. (a) The effect of phenol, guaiacol, furfural, levulinic acid, HMF and vanillin on ethanol concentration**.** (b) The effect of phenol, guaiacol, furfural, levulinic acid, HMF and vanillin on glucose conversion and ethanol yield. Fermentation conditions: 2.0 g/L of phenol, guaiacol, furfural, levulic acid, HMF and vanillin separately tested with 398 g/L glucose at 33 ℃with 160 rpm, approximately 5.0×10^{8} cells/mL, 72 h, and pH of 4.3.

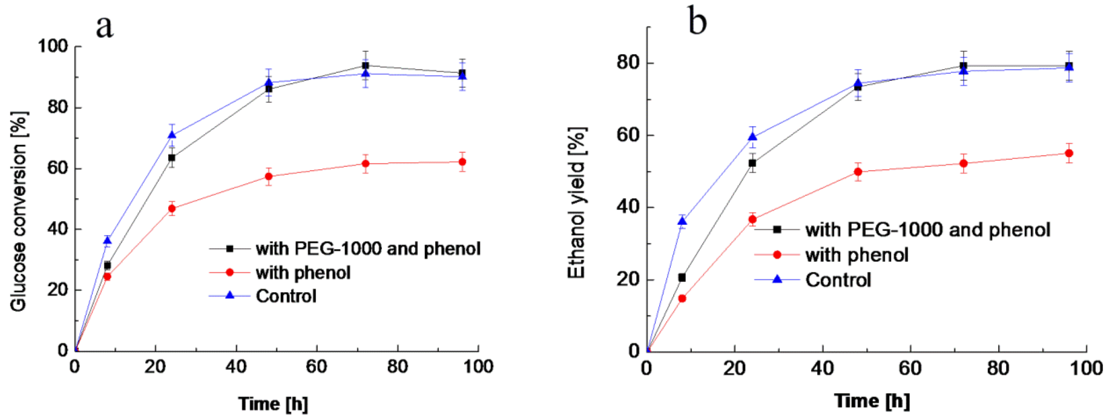

Supplementary Figure S2 Inhibition of phenol on the ethanol production from glucose. (a), Profiles of

glucose conversion during glucose fermentation process. (b), Profiles of ethanol yield during glucose fermentation process. Fermentation conditions: 398 g/L glucose, 2.0 g/L of phenol, approximately 5.0×10^8cells/mL, 0.25mg/L of PEG-1000, 33 ^oC, 160 rpm, and pH of 4.3.

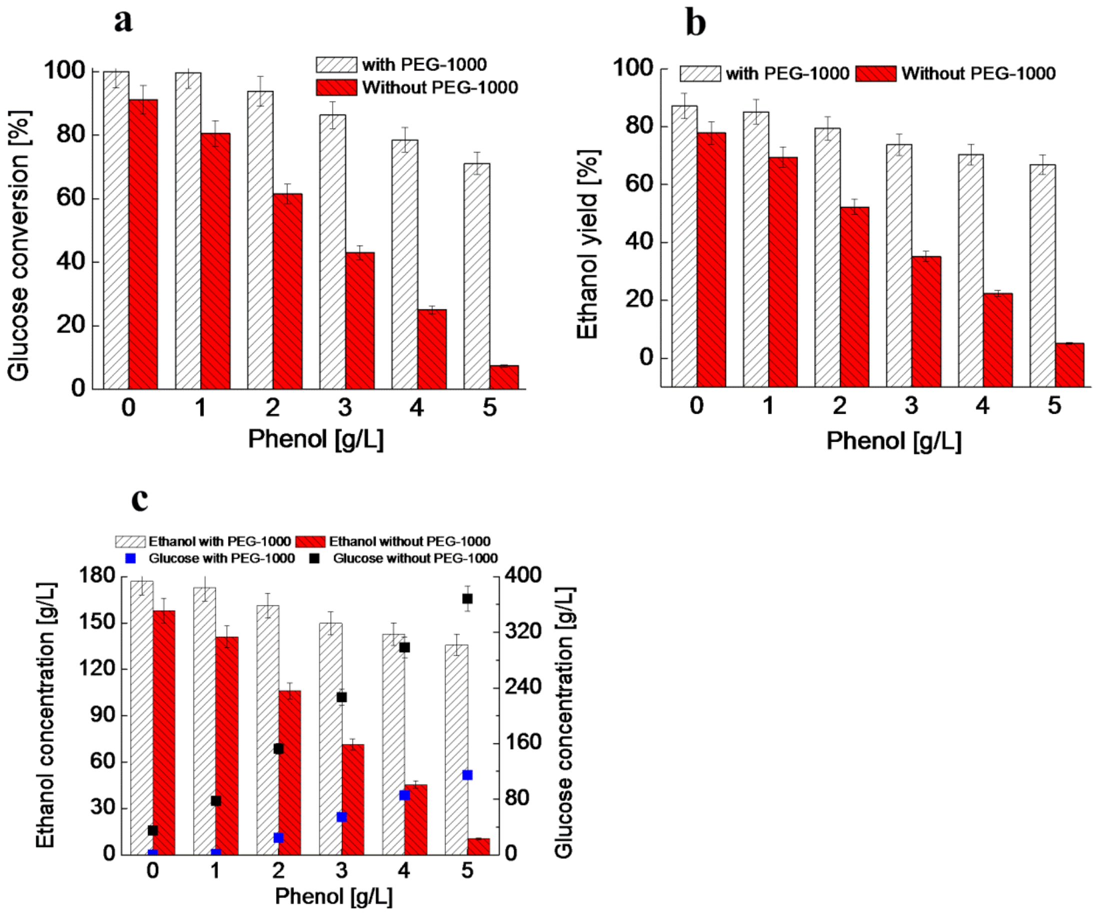

Supplementary Figure S3 The effect of PEG-1000 during fermentation at different phenol concentrations. (a) The effect of phenol concentration on glucose conversion**.** (b) The effect of phenol concentration on ethanol yield. (c) The effect of phenol concentration on ethanol and glucose concentration. Fermentation conditions: 398 g/L glucose, approximately 5.0×10^8cells/mL, 0.25mg/L of PEG-1000, 33 ^oC, 72 h, 160 rpm, and pH of 4.3.

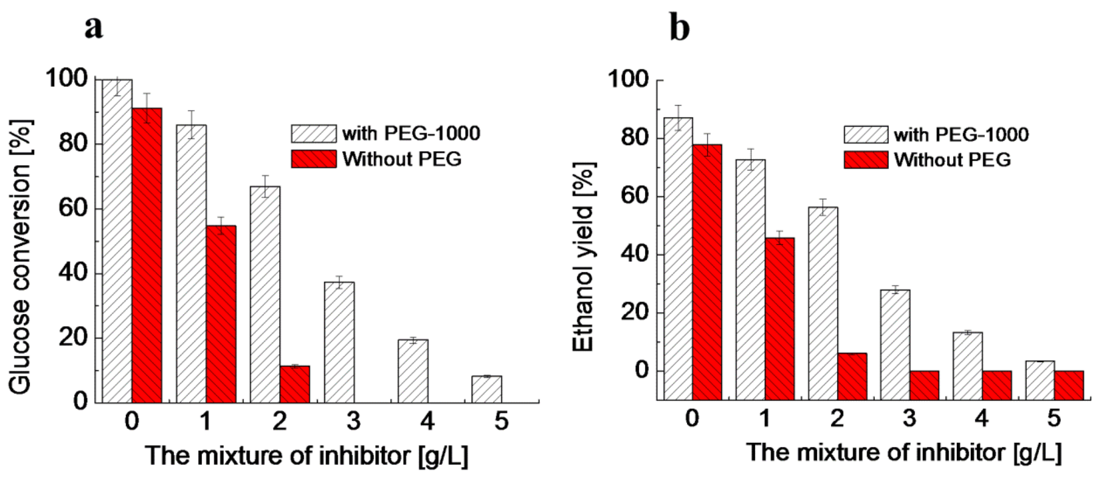

Supplementary Figure S4 The effect of PEG-1000 during fermentation in the presence of mixed inhibitors (a) The effect of PEG-1000 during fermentation in the presence of mixed inhibitors on glucose conversion**.** (b) The effect of PEG-1000 during fermentation in the presence of mixed inhibitors on ethanol yield. Fermentation conditions: 398 g/L glucose, approximately 5.0×10^{8}cells/mL, 0.25mg/L of PEG-1000, 33 ℃, 72 h, 160 rpm, and pH of 4.3.

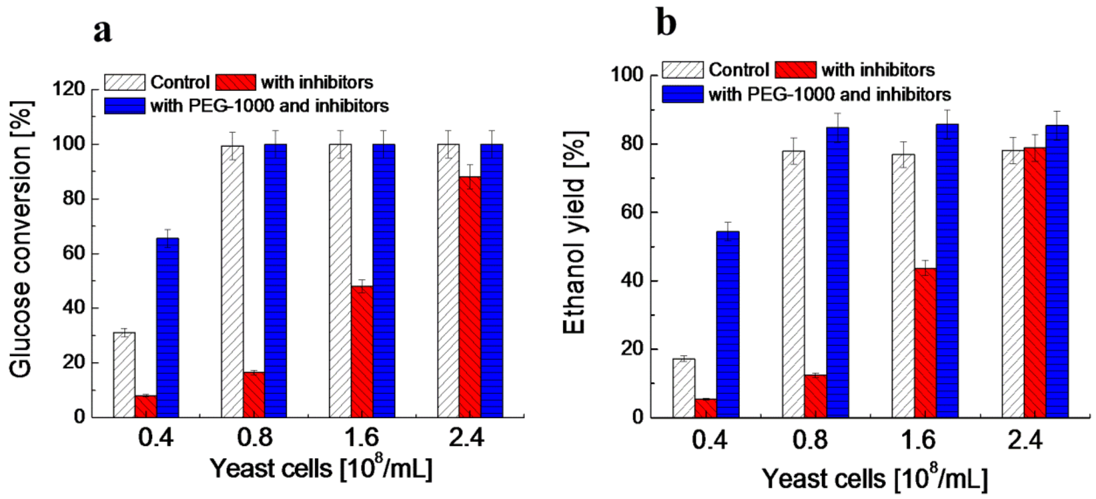

Supplementary Figure S5 The effect of PEG-1000 during ethanol production from low glucose concentration in the presence of mixed inhibitors. (a) The effect of PEG-1000 during ethanol production from low glucose concentration in the presence of mixed inhibitors on glucose conversion. (b) The effect of PEG-1000 during ethanol production from low glucose concentration in the presence of mixed inhibitors on ethanol yield. 72 g/L glucose, 2.0 g/L of inhibitors, 0.2mg/L of PEG-1000, 33 ℃, 48 h, 160 rpm, and pH of 4.3.

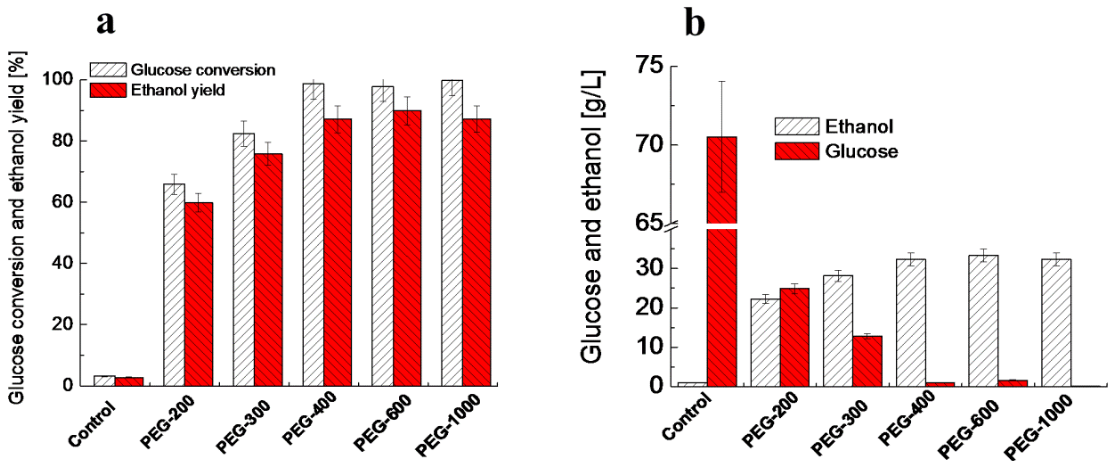

Supplementary Figure S6 Effect of different PEG molecular weight. (a) The effect of different PEG on glucose conversion and ethanol yield**.** (b) The effect of different PEG on glucose and ethanol concentrations**.** Fermentation conditions: 72 g/L glucose, 2.0g/L of inhibitors, 0.2mg/L of PEGs, approximately 0.8×10^{8}cells/mL, 33 ℃, 48 h, 160 rpm, and pH of 4.3.

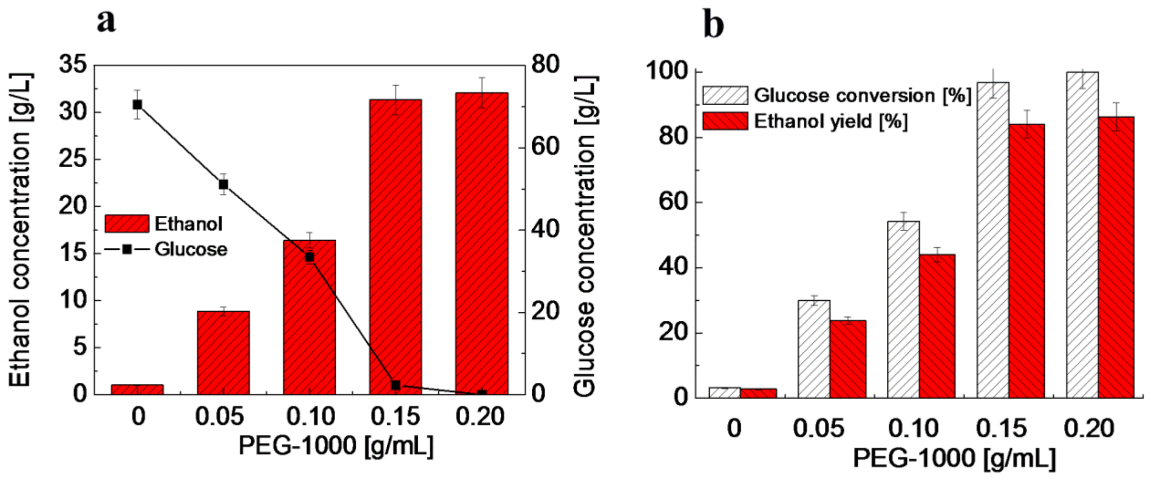

Supplementary Figure S7 Effect of PEG concentration in water. (a) The effect of PEG concentration on ethanol concentration (b) The effect of PEG concentration on glucose conversion**.** Fermentation conditions: 72 g/L glucose, 2.0 g/L of inhibitors, approximately 0.8×10^{8}cells/mL, 33 ℃, 48 h, 160 rpm, and pH of 4.3.

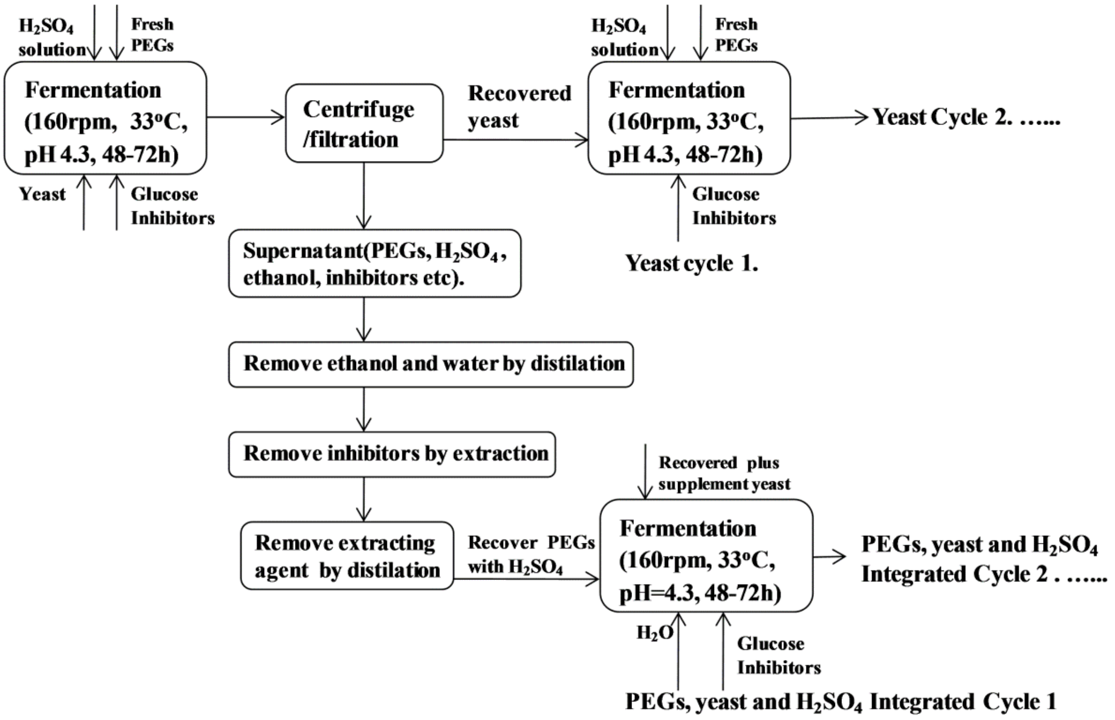

Supplementary Figure S8 Process scheme for the recovery and reuse of yeast, PEGs and H_2SO_4. Experiments on combined reuse of yeast, PEGs and H_2SO_4 followed similar recovery procedure.

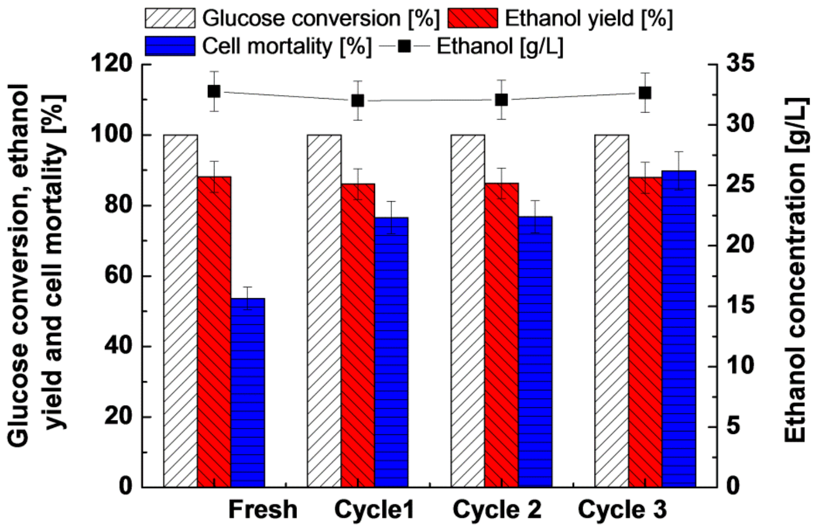

Supplementary Figure S9 Glucose conversion, ethanol yield and concentration, and cell mortality of four successive fermentation runs by recovering yeast, PEG-1000 together with H_2SO_4. Fermentation conditions: 72 g/L glucose, 0.2 g/mL of PEG-1000, 48 h, 33 ℃, 160 rpm, and pH of 4.3.

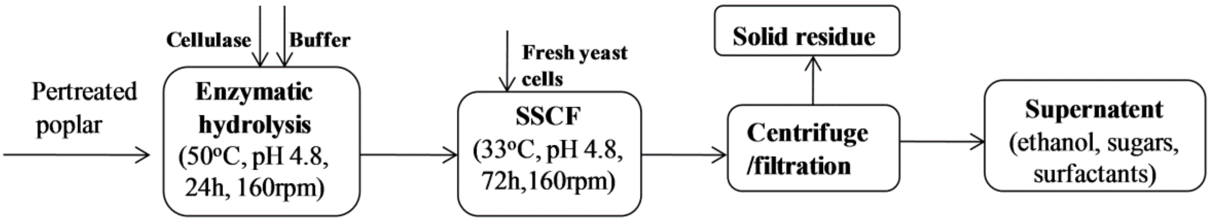

Supplementary Figure S10 Detox SSCF of H&E-poplar
